# Parametric optimisation for the design of gravity energy storage system using Taguchi method

**DOI:** 10.1038/s41598-022-20514-y

**Published:** 2022-11-16

**Authors:** Mostafa E. A. Elsayed, Saber Abdo, Ahmed A. A. Attia, El-Awady Attia, M. A. Abd Elrahman

**Affiliations:** 1 Mechanical Engineering Department, Faculty of Engineering at Shoubra, Benha University, Cairo, 11672 Egypt; 2 Automation and Energy Technology Lab., Mechanical Engineering Department, Shoubra Faculty of Engineering, Benha University, Cairo, Egypt; 3 Combustion and Energy Technology Lab., Mechanical Engineering Department, Shoubra Faculty of Engineering, Benha University, Cairo, 11672 Egypt; 4 Industrial Engineering Department, College of Engineering, Prince Sattam Bin Abdulaziz University, Al Kharj, 11942 Saudi Arabia; 5 Mechanical Engineering Department, University of Bristol, Bristol, UK

**Keywords:** Energy storage, Mechanical engineering

## Abstract

Gravitational energy storage systems are among the proper methods that can be used with renewable energy. However, these systems are highly affected by their design parameters. This paper presents a novel investigation of different design features of gravity energy storage systems. A theoretical model was developed using MATLAB SIMULINK to simulate the performance of the gravitational energy storage system while changing its design parameters. A parametric optimization study was also conducted using Taguchi and analysis of variance (ANOVA) techniques for optimizing the energy storage rate. Six parameters were studied; three are related to the piston design (diameter, height, and material density). The other parameters are the return pipe diameter, length, and charging/discharging time. Results revealed that the piston diameter and height are the two most significant parameters for the system performance compared to the other parameters, as they contributed by 35.11% and 30.28%, respectively. The optimization results indicated that the optimal piston diameter, height, and return pipe diameter were 0.25, 0.5, and 0.01 of the container height. The outcomes of this paper can significantly improve energy storage and power generation from renewable energy systems as it provides a reliable, economical, sustainable, and durable energy storage system.

## Introduction

Renewable energy (RE) generation has increased in recent years and is expected to continue to grow over the coming years. Electricity generated by RE is expected to rise from 10% in 2010 to 35% by 2050^1,2^. However, renewable resources usually cannot be used as a stand-alone power plant or as a primary source of electricity due to their intermittent nature and significant fluctuation, especially wind and solar energy^3,4^. This defect encouraged researchers to develop a solution for this irregular nature. Two immediate solutions have been suggested to address this problem. The first solution is the mixed-use of renewable energy resources, i.e., wind and solar energy. The second is using energy storage devices coupled with renewable energy resources.

There are three critical reasons for storing energy^5–8^; the first reason is transferring power from a non-portable energy source to a portable one. The second is controlling the power-to-energy (PTE) ratio of the energy generation source, which means that the generated output can be directed to meet the changes in energy demand. The last reason is using it later whenever needed to satisfy the increase in demand. An energy storage system that fulfills the second and third reasons can be beneficial in overcoming the intermittent nature of renewable energy. It is worth mentioning that the energy storage systems can also provide flexibility for smart electric grids in the future since they can meet the variation in demand.

Different energy storage systems have been studied and developed over the last two decades. Most of the systems introduced were the electrical, chemical, electrochemical, thermal, and mechanical energy storage^9–11^. Mechanical systems, such as flywheel energy storage (FES)^12^, compressed air energy storage (CAES)^13,14^, and pump hydro energy storage (PHES)^15^ are cost-effective, long-term storage solutions with significant environmental benefits for small- and large-scale renewable energy power plants to overcome energy generation fluctuation^16^.

A relevant study proposed three approaches for combining gravitational storage systems with renewable energy resources^17^. The first was the "Energy Vault Tower", which employs ropes to raise masses using the generated energy. The stored energy can be retrieved by lowering these masses (concrete blocks) while driving an electric generator with ropes^18^. The second method, which can be used in abandoned mine shafts, uses a massive suspended weight rather than multiple concrete blocks^19^. The third method utilizes a heavy piston that moves vertically inside a cylinder by compressing fluid flow through a valve.

According to^20^, the first closed hydraulic circuit was developed by a company called Gravity Power. The main idea was to pump water from a low-pressure side to raise a piston in a closed hydraulic circuit; in this case, this is called the storage phase. When there is a need to recover the stored energy, the piston is allowed to descend by opening a valve, allowing water to flow through a hydraulic turbine and generate electricity. According to Heindl^21^, the efficiency of the round-trip gravitational energy storage system can reach more than 80%.

Gravity storage systems were studied from various perspectives, including design, capacity, and performance. Berrada et al.^22,23^ developed a nonlinear optimization model for cylinder height using a cost objective function. Their findings demonstrated that the Levelized price of gravity energy storage is competitive with other techniques. Furthermore, the proposed small-scale gravity storage systems could be stand-alone renewable energy storage systems. Berrada et al.^24^ also numerically examined the use of various materials in gravity storage systems. They suggested using “iron ore” for the piston and reinforced concrete for the system container.

On the other hand, valuable efforts were made to avoid the use of heavy pistons and improve system performance^25^. Botha and Kamper^26^ investigated a waterless gravity energy storage system with a wire rope hoist and drive train technology up to 90% efficiency^27,28^.

Statistical analysis of energy storage systems should be considered as they reduce experimental costs, which helps minimize the research cost and time. It also offers a comprehensive view of parameters influencing the system performance^29^. In a relevant study, Elsayed et al.^30^ added a fuzzy control system to a gravity energy storage system, employing three fuzzy membership functions, triangular, trapezoidal, and Gaussian, to determine the appropriate design parameters criteria for various sized power plants. Their results showed that the Gaussian membership function best represents the fuzzy model of the storage system.

Statistical methods are also necessary to improve the forecasting and management of supply and demand in energy storage operations. Rehman et al.^31^ used recent advances in deep learning methods and compared them with traditional statistics to forecast hourly natural gas demand in five locations in Spain. They concluded that the benefits and drawbacks of both classical and deep learning methods should be considered before deciding on a suitable technique. Traditional methods can outperform deep learning methods when prediction accuracy is highly weighted. Deep learning methods may be a good option if avoiding extreme/negative predicted values is very important^32,33^. Generally, there are two optimization strategies; traditional and statistical. The traditional optimization strategy is based on changing one parameter while all other parameters remain constant. The main disadvantage of the traditional method is being time-consuming and costly.

On the other hand, the statistical design of experimental methods provides a straightforward and equally efficient approach. The evolutionary operation, factorial, regression, response surface, and Taguchi methods are the most used for experimental design^34–36^. Ibrahim et al.^37^ presented Taguchi optimization of tribological behaviors of composite materials. They concluded that Taguchi and analysis of variance (ANOVA) techniques are promising for predicting tribological behavior and can then be used to guide the design and implementation of tribological materials.

Taguchi's method is superior to other optimization methods because it allows simultaneous optimization of multiple factors. Furthermore, fewer experimental trials can yield more quantitative information. Taguchi's method has been used in various fields, including renewable energy generation and energy storage systems^38–41^.

The primary literature demonstrates that the capacity of gravity energy storage can be increased by selecting appropriate geometrical design parameters. Furthermore, hydraulic losses can be reduced by efficiently designing the system's components and selecting suitable devices. As a result, more investigation is needed to understand and optimize the parameters affecting the performance of gravity storage systems. This study investigates various design parameters that can affect the performance of a small-scale gravity storage system. It also presents a comprehensive model to optimize these design parameters. Six system design parameters are investigated, including three piston-related parameters (diameter, height, and density), in addition to three other parameters related to system components; return pipe diameter, length, and charging/discharging time.

This paper presents a novel comprehensive model that predicts and optimizes the most influencing parameters on the performance of gravitational energy storage systems. The simulated model using MATLAB-SIMULINK was created and validated against experimental data from the literature before applying the statistical approach. The Taguchi method was then used to predict the contribution of design parameters to system performance and to determine the best combination of parameters to maximize system performance due to its simplicity and dependability.

## Research methodology

**Figure 1:**
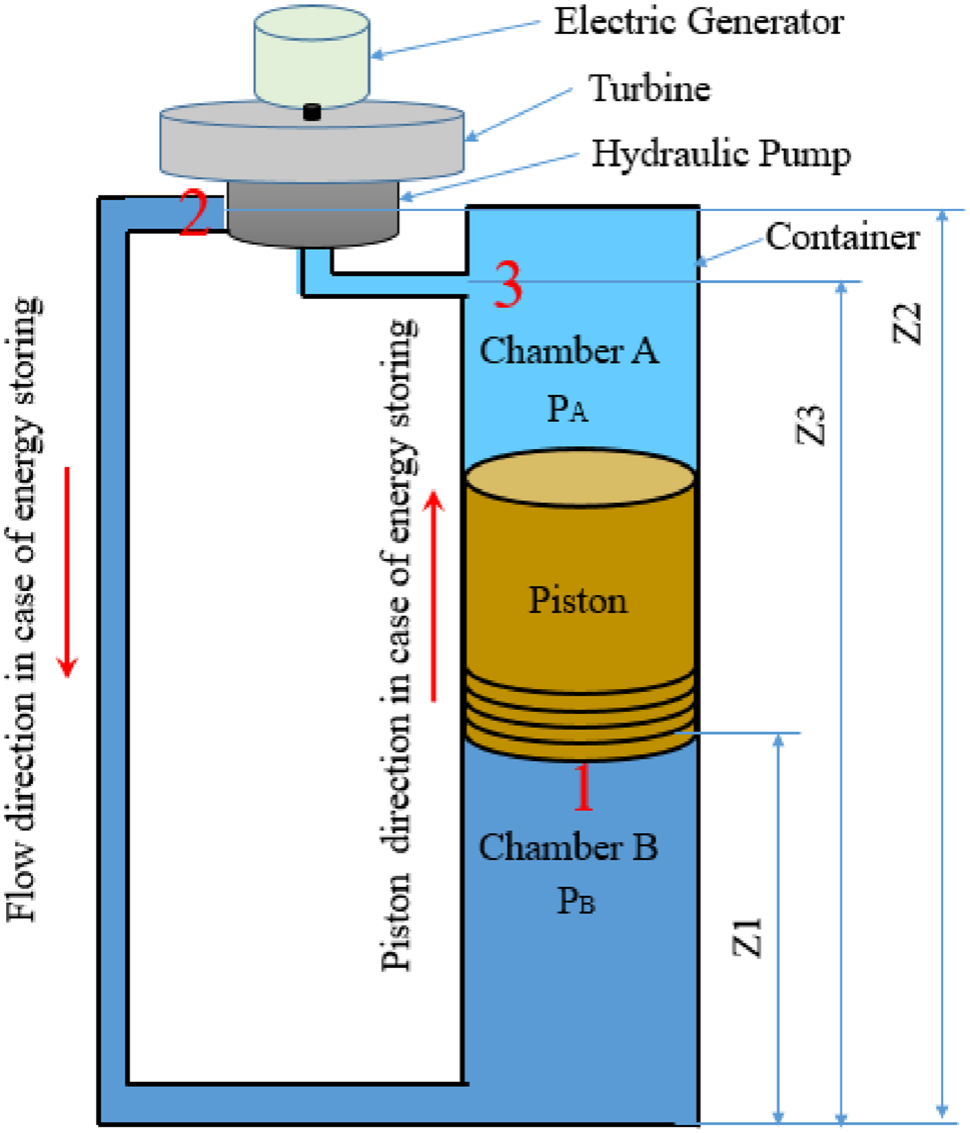
Schematic illustration of gravity energy storage.

Figure 1 shows the general components of the gravity storage system investigated in this study. There are two main working cycles in these systems. The first is the charging phase, where a pump uses the available electricity to store a pressurized liquid in chamber B with a heavy-weight piston on the top; the pump pushes the fluid from point 3 to point 1. The second phase is the discharging phase, in which the piston weight drives the flow from point 1 to point 2 while the pump works as a hydraulic turbine. This process uses different flow control valves to manage the charging and discharging rates.

This research was divided into six stages. The first stage was performing the mathematical modelling of the system by applying the governing equations. The second stage was the development of a virtual simulation of the system using MATLAB/Simulink. This simulation is used to investigate the system performance.

**Figure 2:**
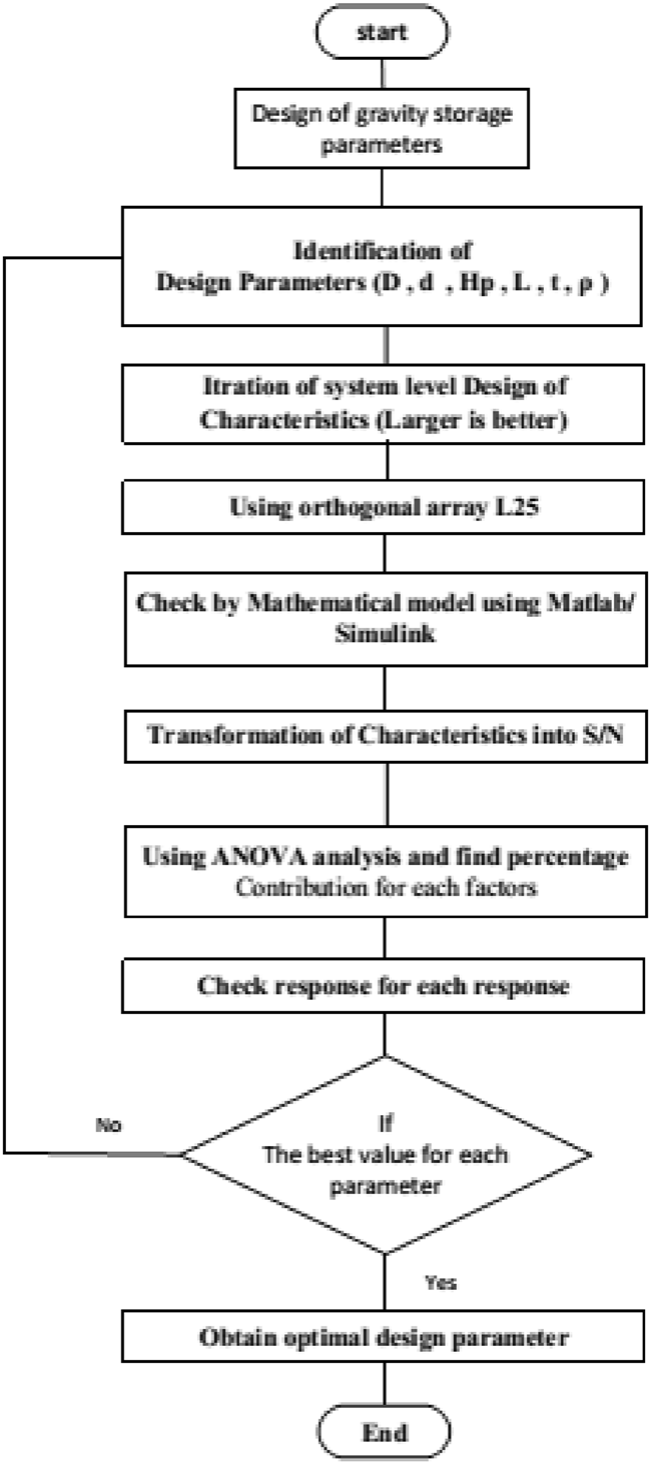
Flow chart for the presented algorithm.

The third stage was the model validation against existing experimental work from the literature. The fourth is the preliminary analysis used to investigate the effect of the different design parameters on system performance. The fifth step is the design of the experiment (DOE) based on the Taguchi method and obtaining different levels of the parameters for each experimental trial. The levels of parameters were used to optimize the system performance using the simulation model. The final step was the statistical analysis using Taguchi signal-to-noise analysis and the ANOVA analysis. The optimal combination of the design parameters was identified and discussed based on the statistical results. The flow chart of the present algorithm is shown in Fig. 2.

### Mathematical modelling and simulation

The equations describing the systems are applied to numerically investigate the parameters that can significantly affect a gravity energy storage system. As there are different interactions between system components, the motion of the model was built by adopting Berrada et al. in^22,30^ technique, with some modifications over the main assumptions and strategies.

- The initial volume in chamber A, $V_{A,0} { } = { }0$; i.e., empty means the system is fully charged.
- The initial volume contained in chamber B, $V_{B,0} = \left( {H_{C} - H_{P} } \right)A_{B}$, where $H_{C} \;and\;H_{P}$ are the chamber and the piston heights, respectively; initially, all the liquid is below the piston.
- Applying the mass conservation equation on the system as the piston starts to move a distance $x_{p}$. Assuming that the system's volume would not change with the operation and ideal conditions where there is no leakage in the design, the contained volumes at points A and B were calculated using Eqs. (1) and (2), respectively.
  1 $$V_{A} = V_{A,0} + x_{p} \frac{{\pi D^{2} }}{4}$$
  2 $$V_{B} = \left( {H_{C} - H_{P} } \right)V_{B,0} - x_{p} \frac{{\pi D^{2} }}{4}$$

- where D is the piston diameter in (m).

- Applying the continuity equation between chambers A and B gives:
  3 $$\dot{m}_{A} = - \dot{m}_{B}$$
  4 $$\rho \dot{V}_{A} = \rho \dot{V}_{B}$$
  where
- $\dot{m}$: is the mass flow rate in kg/s.
- $\rho$: is the fluid density in kg/m^3^.
- $\dot{V}$: is the volume flow rate in m^3^/s.

As the system is operating at high pressure, the fluid density difference should be considered as there will be energy used in such a system to compress the fluid. The density, as a function of the system's pressure, was estimated using Eq. (5) as follows:

5 $$\rho = E \frac{\partial \rho }{{\partial P}}$$

where P: is the system pressure in Pa.

E: is the bulk modulus of the fluid at the given pressure that was calculated using the following equation^42^:

6 $${\text{E}} = \frac{1}{2} \,E_{0} \;\log_{10} \left( {K_{1} \frac{P}{{P_{o} }} + K_{2} } \right)$$

where

- $E_{0}$: The fluid bulk modulus at one atmospheric pressure.
- $P_{0}$: The standard pressure of 1 atmospheric pressure.
- $K1\;and\;K2$: Empirical constants of 90 and 3. (Assuming isothermal case).

Combining the continuity equation with the variation of bulk modulus with the pressure change, the pressure change in chambers A and B are expressed as:

7 $$\frac{{dP_{A} }}{dt}{ } = \frac{E}{{V_{A} }}{ }\left( {\dot{V}_{A} { } - { }A\frac{{dx_{P} }}{dt}} \right)$$

8 $$\frac{{dP_{B} }}{dt}{ } = - { }\frac{E}{{V_{B} }}{ }\left( {\dot{V}_{B} - { }A\frac{{dx_{P} }}{dt}} \right)$$

After obtaining the pressure as a driving force for the system, Newton's second law was applied as follows:

9 $$\sum F = m\ddot{X}_{p} = P_{A} A_{A} - P_{B} A_{B} + mg - f_{f}$$

where $f_{f}$ is the friction force between the piston and the chamber walls, which is a function of the sealant material that is equal to

10 $$f_{f} = { }\mu N$$

where $\mu$: is the friction coefficient estimated as 0.1 and N is the normal force exerted by the sealant on the walls calculated using Eqs. (12–16) in^43^.

Rearranging Eq. (9) for the acceleration of the piston ($\ddot{X}_{p}$)

11 $$\ddot{X}_{p} = \frac{1}{m}\left( {P_{A} A_{A} - P_{B} A_{B} + mg - \mu N} \right){ }$$

To avoid the problems associated with the extreme positions of the piston, the logic function shown in Eq. (12) was used for assigning the acceleration condition.

12 $${\text{If}}\left\{ {\left( {X_{P} \ge X_{min} \;\& \;a_{P} < 0} \right)\;{\text{or}}\;\left( {X_{P} \le X_{max} \;\& \;a_{P} > 0} \right)} \right\}\;{\text{then}}\;a_{p} = = ,\;{\text{else}}\;a_{p} = = \ddot{x}_{P}$$

As the hydraulic energy stored and recovered from the system will be proportionally associated with the hydraulic losses in the design, it was crucial to consider the fluid friction in the pipes. The hydraulic losses related to the fluid flow in the system are estimated using the Darcy-Weisbach equation and the Moody chart^43^.

Reynolds number was determined using Eq. (13) as follows:

13 $$Re = \frac{\rho vd}{\mu }$$

where

- v: is the fluid flow velocity in (m/s).
- d: is the flow diameter in (m).
- $\mu$: is the fluid viscosity in Pa.s.

Then the major hydraulic losses ($h_{L,major}$) are estimated using the Darcy-Weisbach equation that is expressed by Eq. (14) in the function of frictions $f_{1}$ and $f_{2}$.

14 $$h_{L,major} = \frac{{0.0827 \times f_{1} \times x_{p} \times \dot{V}^{2} }}{{D^{5} }} + \frac{{0.0827 \times f_{2} \times L_{pipe} \times \dot{V}^{2} }}{{d^{5} }}$$

The friction coefficients $f_{1}$ and $f_{2}$ are for the piston and the pipe, respectively. They were estimated separately using their diameters from Eq. (15):

15 $$f_{1,2} = \frac{1.325}{{\left[ {ln\left( {\frac{{0.045 \times 10^{ - 3} }}{{3.7{ }d^{2} }} + \frac{{1.5 \times 10^{ - 5} { } \times d^{0.9} }}{{{ }\dot{V}{ }^{0.9} }}} \right)} \right]^{2} }}$$

Furthermore, the minor losses were assumed to be 50% of the friction losses owing to the path's numerous bends. Consequently, the hydraulic losses were calculated using Eq. (16).

16 $$h_{l, total} = 1.5 h_{L,major} \quad ({\text{m}})$$

Bernoulli equation was applied between point 1 and 2 to calculate the hydraulic head created by the pump, as follow:

17 $$\frac{{P_{1} }}{\rho g} + \frac{{V_{1}^{2} }}{2g} + Z_{1} + H_{pump} - { }H_{hyd - turbine} = { }\frac{{P_{2} }}{\rho g} + \frac{{V_{2}^{2} }}{2g} + Z_{2} + h_{l,total}$$

The inlet pressure $P_{1}$ can be calculated by applying the Bernoulli equation between the inlet (1) and chamber B as expressed by Eq. (18).

18 $$\frac{{P_{1} }}{\rho g} = \frac{{P_{B} }}{9810} + 0.0827\dot{V}^{2} { }\left( {\frac{1}{{D^{4} }} - \frac{1}{{d^{4} }}} \right) + \left( {Z_{1} - Z_{2} } \right) - h_{{l,{ }total{ }1 - B}}$$

Then the outlet pressure $P_{2}$ can be calculated by applying the Bernoulli equation between the outlet (2) and chamber A as expressed by Eq. (19).

19 $$\frac{{P_{2} }}{\rho g} = \frac{{P_{A} }}{9810} + 0.0827\dot{V}^{2} { }\left( {\frac{1}{{D^{4} }} - \frac{1}{{d^{4} }}} \right) + \left( {Z_{1} - Z_{2} } \right) - h_{{l,{ }total{ }2 - A}}$$

Finally, the hydraulic power generated by the turbine was calculated using Eq. (20) as follows:

20 $$P_{hyd - turbine} = H_{hyd - turbine} \times 9810 \times \dot{V}$$

where

- $P_{hyd - turbine}$: is the hydraulic power available for the turbine in (W).

The governing equations of a gravitational energy storage system were used to develop a Simulink model on MATLAB software^30^.

### Model validation

A model was created using Matlab/Simulink and was validated against the experimental results reported in^44^. This reference was chosen as experimental data were available for comparison^44^. The physical model data are presented in Table 1.

**Table 1:** Reference data of the physical model.

Container height $\left( {H_{c} } \right)$, m	Piston diameter $\left( D \right)$, m	Piston height $\left( {H_{p} } \right)$, m	Piston density $\left( {\rho_{p} } \right)$	Piston mass (kg)	Charge/discharge time (t), min	Return pipe diameter (d), m	Return pipe length (L), m	Return pipe length (L), m
2.2	0.6	0.67	7850	1500	6	0.025	2	2

The data shown in Table 1 were used as input parameters for the model and then compared with the actual results reported in^30,44^. After completing the validation process, different parameter ranges were chosen to investigate their effect on the energy storage performance. Table 2 presents the stimulated ranges used in this study. On the basis of the reference model, these ranges were selected to investigate the effect of increasing and decreasing the design parameters. As the container height is the most significant design parameter, it will be considered as a fixed value, while the others are modified as follows:

**Table 2:** Simulated parameters ranges.

Investigated parameter	The simulated range
Piston diameter/container height ratio D/H_c_	0.05–0.25
Piston height/container height ratio Hp/H_c_	0.1–0.5
Return pipe diameter/container height ratio d/H_c_	0.01–0.02
Return pipe length/container height ratio l/H_c_	0.5–1.5
Charging/discharging time (min)	6–30
Piston specific density	2.7–12

## Results and discussion

According to the given ranges in Table 2, the effect of each parameter as a function of the container height is determined. Then, the output values of the Taguchi method were used to investigate the most and the least significant parameters in the design of the gravity storage system. While changing one of the parameters in this section, all other parameters are considered unchanged from the reference values given in Table 1 above.

**Figure 3:**
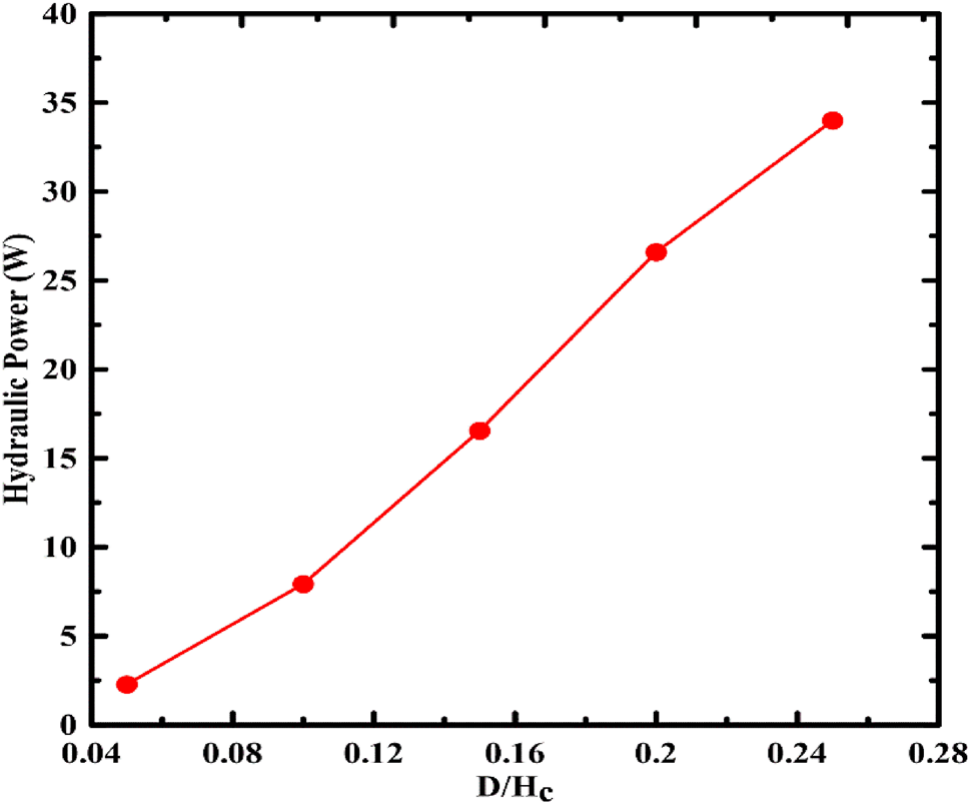
Hydraulic power versus diameter to container height ratio.

The first studied parameter was the piston diameter to container height ratio (D_p_/H_c_). Five values (0.05, 0.1, 0.15, 0.2, and 0.25) were simulated versus the energy stored in the system. Figure 3 presents the output power versus the piston diameter/container height ratio change. From the figure, it can be seen that the hydraulic power increases with increasing the piston's diameter. This increase is a result of increasing the weight of the piston as the piston and fluid volume increase. The rise in the fluid volume increases the flow rate considering that the discharge time is constant at 6 min.

**Figure 4:**
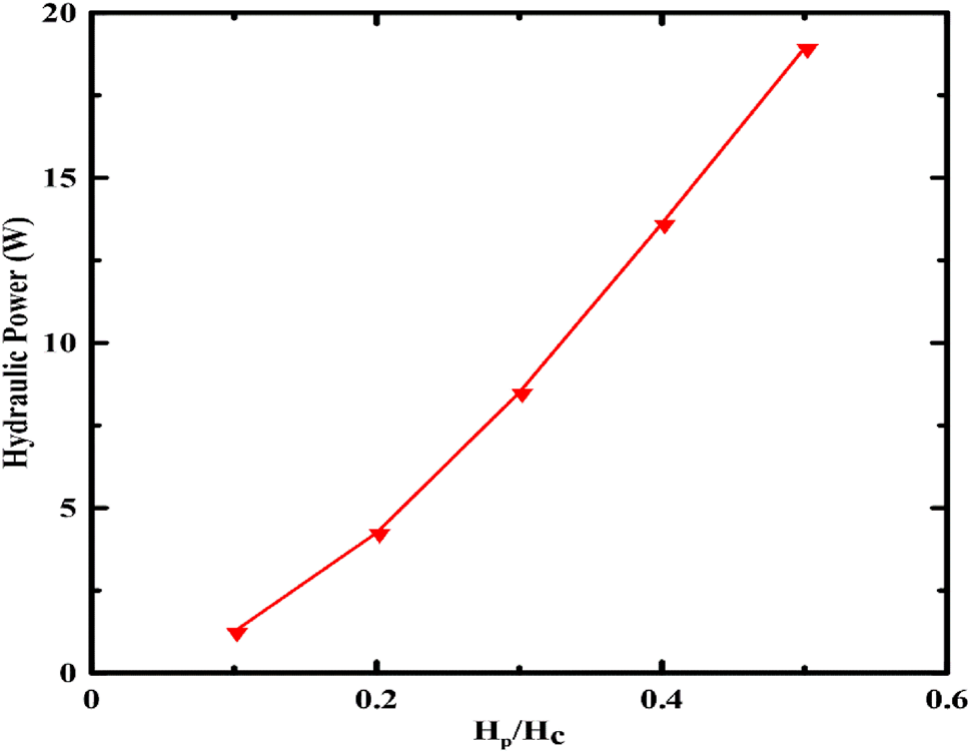
Effect of piston height variation on the hydraulic power.

The second parameter was the piston height to container ratio (H_p_/H_c_) (Fig. 4). The range of 0.1–0.5 was selected with a step of 0.1. It can be noticed from the chart that the hydraulic power and the piston height are directly proportional due to the effect of increasing the height on the piston weight and the pressure difference.

**Figure 5:**
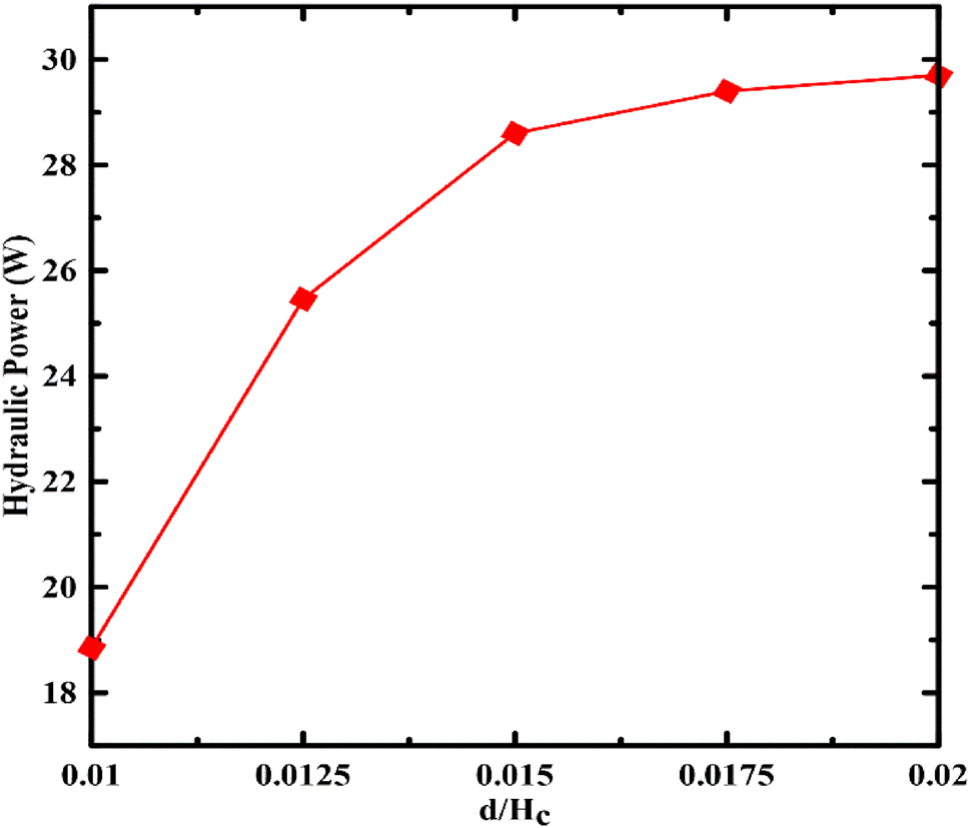
Effect of return pipe diameter variation on the system's power.

For the return pipe, two design factors are considered: the pipe diameter (d) and the pipe length (L). Figure 5 represents the influence of return pipe diameter (d) on the storage power. As the return pipe diameter to container height grows from 0.01 to 0.02, the resultant energy steadily climbs to the highest value at the highest diameter ratio of 0.02. This occurred because of the fact that increasing the diameter of the discharge pipe lowers the flow rate and, consequently, the friction losses of the pipe.

**Figure 6:**
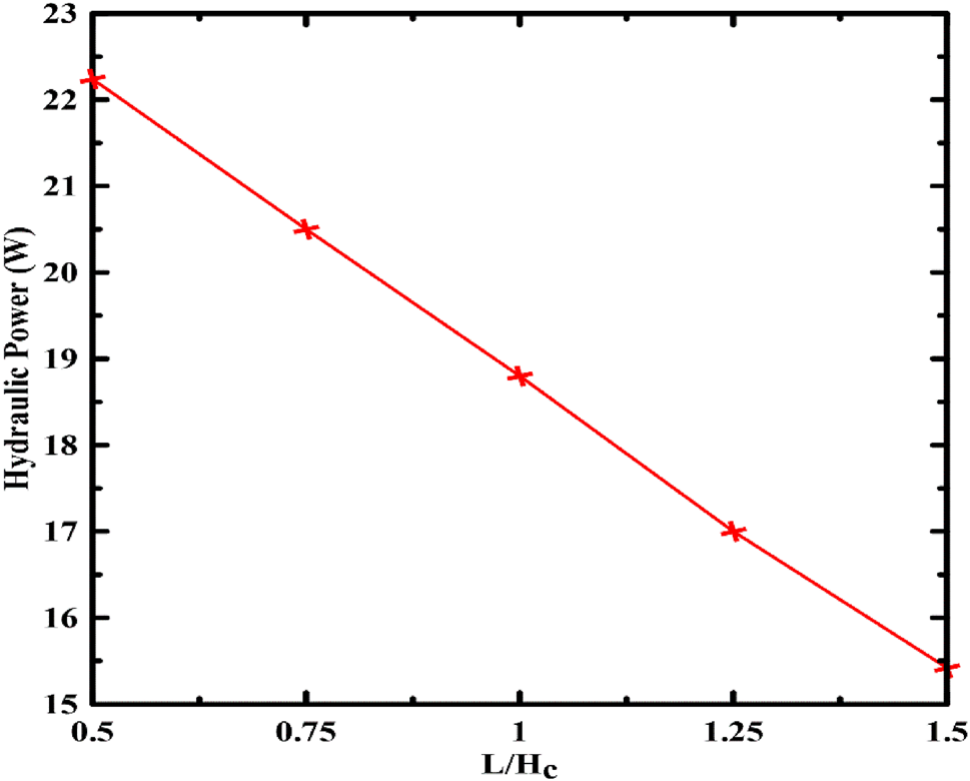
Effect of return pipe length variation on the hydraulic power.

Figure 6 illustrates the influence of return pipe length on the power generated. Within the studied range between a ratio of 0.5–1.5, it can be shown that increasing the length increases the friction losses and, thus, reduces the available hydraulic power.

**Figure 7:**
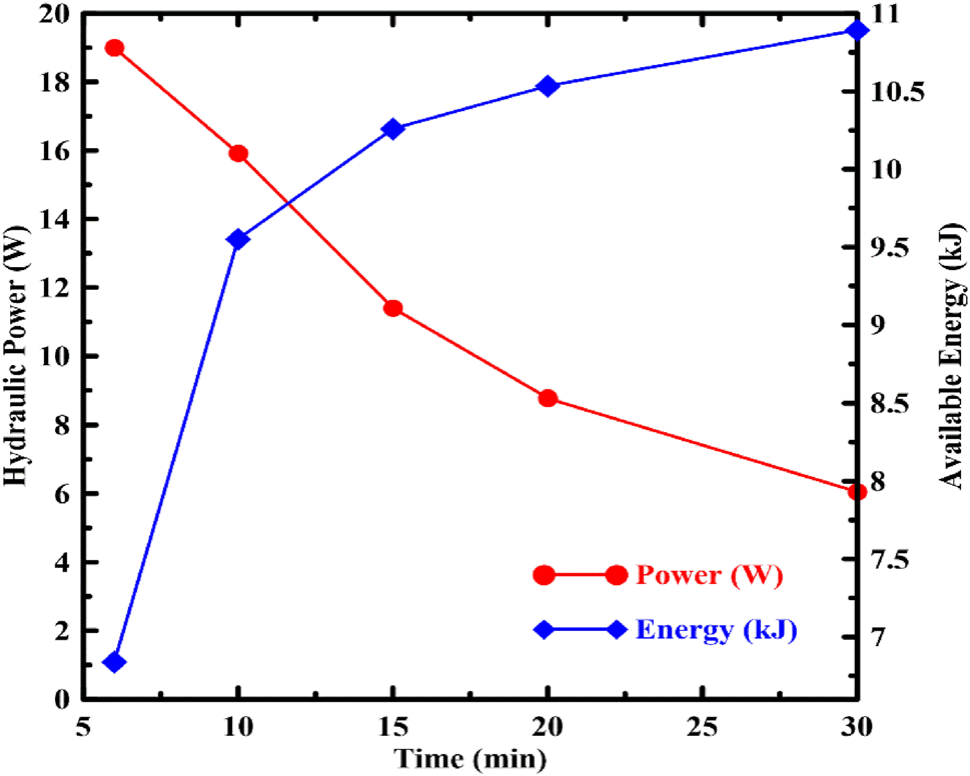
Effect of charge/discharge time variation on the power.

The output power and available energy extracted from the system at different charge/discharge times are shown in Fig. 7. As the charge/discharge time rises from 6 to 30 min, the resulting energy gradually increases to the highest value at 30 min. This is due to the increase in the discharge time slows down the volume flow rate and discharge speed. Reducing the discharging speed significantly reduces the hydraulic losses and increases the available power.

**Figure 8:**
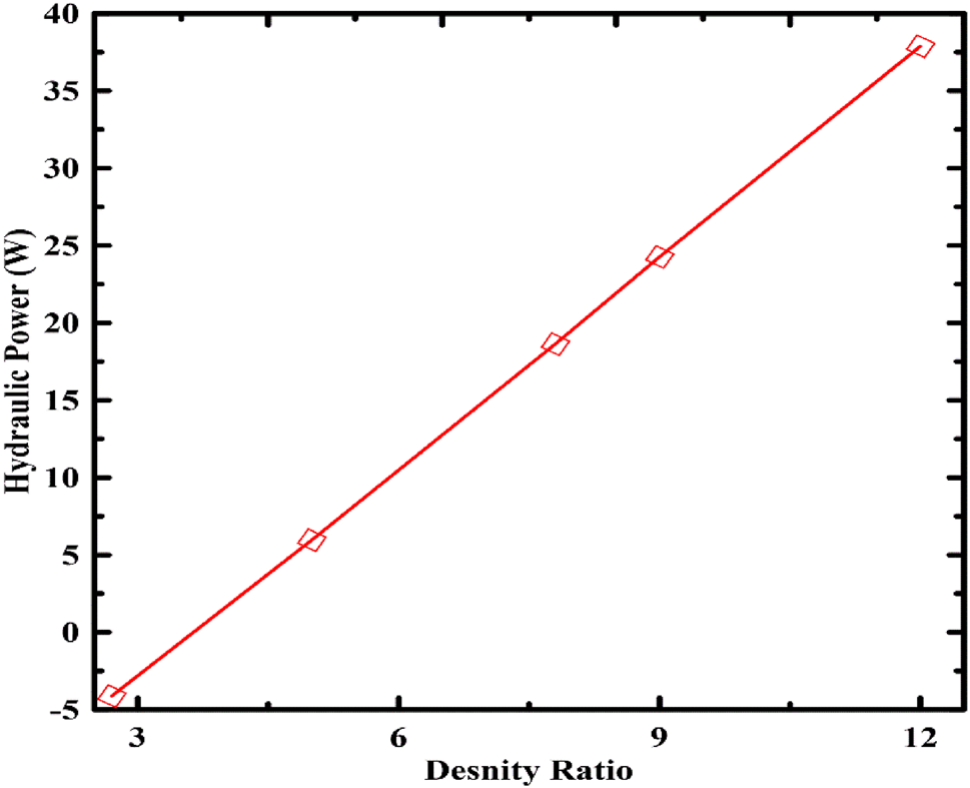
Effect of density ratio variation on the system's power.

Figure 8 shows the influence of the piston density ratio on the power. It can be observed that when the density ratio of the piston to water increases from 2.7 to 12, The resultant power progressively climbs to the most significant value at the density ratio of 12. As the piston density increases, the piston weight increases, giving a higher pressure difference that increases the system's available power.

The second step in the current research was to investigate the effect of the design parameters with varying container heights. The container height is varied over the range between 2.2 and 20 m. The interaction between design parameters is shown in Tables 3, 4, 5, 6, 7 and 8. Each table shows the resultant generated power considering one variable from the design parameters at different container heights.

**Table 3:** Interaction effect of D and Hc.

D/Hc	Hc				
	2.2	5	10	15	20
0.05	1.88	50.1	799	4033	12,706
0.1	7.54	190	2876	**13,693**	**40,534**
0.15	16.46	**330.6**	**3336**	7002	− 9123
0.2	26.84	118.9	− 9074	− 101,503	− 496,411
0.25	**33.95**	− 1359	− 63,617	− 534,028	− 2,357,703

Significant values are in bold.

**Table 4:** Interaction effect of Hp and Hc.

Hp/Hc	Hc				
	2.2	5	10	15	20
0.1	1.12	2.01	32.15	162.6	513.7
0.2	1.2	31.5	493	2441	7539
0.3	5.8	136.6	**1764**	**6793**	**14,718**
0.4	**13.5**	**163.8**	− 2998	− 43,625	− 227,789
0.5	3.98	− 1359	− 63,617	− 534,028	− 2,357,703

Significant values are in bold.

**Table 5:** Interaction effect of d Hc.

d/Hc	Hc				
	2.2	5	10	15	20
0.01	18.8	118.8	− 9074	− 101,503	− 496,411
0.0125	26.5	580.1	5684.4	10,569.9	− 24,138
0.015	28.7	714.5	9986.8	43,240.9	113,536
0.0175	29.5	763.1	11,540.1	55,036.3	163,242
0.02	**29.8**	**783.4**	**12,191.5**	**59,983.3**	**184,088**

Significant values are in bold.

**Table 6:** Interaction effect of Lpipe and Hc.

Lpipe/Hc	Hc				
	2.2	5	10	15	20
0.5	**22.2**	**324.6**	− 2488	− 51,494	− 285,676
0.75	20.6	221.8	− 5781	− 76,498.3	− 391,044
1	18.8	118.9	− 9074	− 101,503	− 496,411
1.25	17	15.9	− 12,366	− 126,507	− 601,779
1.5	15.5	− 86.9	− 15,659	− 151,511	− 707,147

Significant values are in bold.

**Table 7:** Interaction effect of piston relative density and Hc.

Density ratio	Hc				
	2.2	5	10	15	20
2.7	− 4.1	− 494.8	− 18,892	− 151,209	− 653,509
5	6	− 218	− 14,464	− 128,792	− 582,661
7.8	18.8	118.8	− 9074	− 101,503	− 496,411
9	24.3	263	− 6763	− 89,806	− 459,447
12	**37.8**	**624**	− 988	− 60,567	− 367,037

Significant values are in bold.

**Table 8:** Interaction effect of charging/discharging time and Hc.

Time	Hc				
	2.2	5	10	15	20
6	**18.8**	118.8	− 9074	− 101,503	− 496,411
10	15.6	**334.7**	2984	3109	− 28,105
15	11.3	278	**3746**	**15,408**	37,459
20	8.7	222.9	3270	15,056	**42,845**
30	5.9	155	2399	11,704	35,588

Significant values are in bold.

Table 3 shows the results obtained after using variable piston diameters versus container heights. It can be seen that increasing the container height can significantly increase the generated power while increasing the stored potential energy. The limitation applied to this case will be determined according to the installation location parameters.

Table 4 depicts the interaction effect of piston height and container height on the generated power. It can be seen that the optimum piston height ratio is 0.4 Hc for container heights less than 5 m, while the best ratio drops to 0.3 Hc for container heights more than 20 m. This change occurred due to the influence of the volume height on the stored fluid capacity and its flow rate, as well as the increased pressure created by utilizing pistons with greater heights.

The data of the interaction between the return pipe diameter and container height on the system power are tabulated in Table 5. Increasing the discharge pipe diameter can increase the power generated as the losses in that pipe decrease in all of the studied cases. The optimal pipe diameter ratio of 0.02 Hc remains constant while the container height grows from 2.2 to 20 m.

Table 6 shows the influence of the return pipe length ratio on storage power at various container heights. The results reflected that the smaller the pipe length, the more efficient the system would be. This is because of the reduction in the losses of the discharge pipes. For higher container heights, as the length is taken as a function of height, the losses caused were higher than the generated power, so these values are not recommended for the actual case applications due to their inefficiency.

In Table 7, piston density ratios versus piston heights are presented. The data showed that the optimal piston density ratio of 12 remains constant as the container height grows from 2.2 m to 20 m; however, after the height of 5 m, there is a negative effect on the system performance due to the massive increase in system's weight that causes the flow rate and system losses to increase.

Table 8 shows that the optimal charging/discharging time increases with increasing the container height due to the need to reduce the flow velocity to minimize the friction losses in the discharging pipe.

Based on the previous analysis, it can be concluded that the system's design parameters significantly affect its performance. Consequently, the effect of these parameters should be investigated and optimized. An optimization study for different influencing parameters was done using the Taguchi method.

In the following, a trial is performed to estimate the optimum threshold for each design parameter for maximum power output and determine the most significant control parameters of the system^45,46^.

The Taguchi approach yields an orthogonal array as an experimental design. This array contains a series of experiments created by combining different parameter values known as control variables. The control variables and their values should be initially specified in the Taguchi analysis. Optimizing the efficiency of the gravity energy storage system yields hydraulic power. Using Taguchi analysis, six control variables representing the design parameters are defined to optimize the stored energy. Based on the previous preliminary investigation, the range of each factor and its levels can be given as listed in Table 7. The levels will be used to produce the DoE for the analysis. The current study considered five levels for each parameter listed in Table 9.

**Table 9:** Design and operating parameters and their levels in Taguchi DoE.

Parameters		Levels				
Label	Description	1	2	3	4	5
A	D/Hc	0.05	0.1	0.15	0.2	0.25
B	L/Hc	0.5	0.75	1	1.25	1.5
C	d/H	0.01	0.0125	0.015	0.0175	0.02
D	Density ratio	2.7	5	7.8	9	12
E	Hp/Hc	0.1	0.2	0.3	0.4	0.5
F	Time (min)	6	10	15	20	30

The Taguchi design is produced using Minitab 19 software, as shown in Table 10. An orthogonal array of L25 is constructed. The simulation model runs the different trials and obtains the corresponding system output. Only one response is considered at a time. Moreover, the response is represented as values of a potential hydraulic power that the system can store. The result of the simulation for each trial is listed in Table 10.

**Table 10:** Taguchi design of L25 orthogonal array.

Number of trials	Factor A	Factor B	Factor C	Factor D	Factor E	Factor F	P-Hyd	S/N
	D/Hc	L/Hc	d/H	Density ratio	Time	Hp/Hc		
1	0.05	0.5	0.01	2.7	6	0.1	0.1516	− 16.38
2	0.05	0.75	0.0125	5	10	0.2	0.4207	− 7.5213
3	0.05	1	0.015	7.8	15	0.3	0.6338	− 3.9613
4	0.05	1.25	0.0175	9	20	0.4	0.6407	− 3.8665
5	0.05	1.5	0.02	12	30	0.5	0.6142	− 4.2343
6	0.1	0.5	0.0125	7.8	20	0.5	2.2628	7.0929
7	0.1	0.75	0.015	9	30	0.1	0.6402	− 3.8739
8	0.1	1	0.0175	12	6	0.2	7.8422	17.8888
9	0.1	1.25	0.02	2.7	10	0.3	0.9018	− 0.8976
10	0.1	1.5	0.01	5	15	0.4	1.6681	4.4445
11	0.15	0.5	0.015	12	10	0.4	15.8794	24.0167
12	0.15	0.75	0.0175	2.7	15	0.5	1.6098	4.1357
13	0.15	1	0.02	5	20	0.1	1.0619	0.5220
14	0.15	1.25	0.01	7.8	30	0.2	1.1507	1.2194
15	0.15	1.5	0.0125	9	6	0.3	15.6611	23.8964
16	0.2	0.5	0.0175	5	30	0.3	2.9424	9.3739
17	0.2	0.75	0.02	7.8	6	0.4	28.7657	29.1775
18	0.2	1	0.01	9	10	0.5	21.2950	26.5656
19	0.2	1.25	0.0125	12	15	0.1	6.2517	15.9200
20	0.2	1.5	0.015	2.7	20	0.2	1.2849	2.1771
21	0.25	0.5	0.02	9	15	0.2	14.1279	23.0015
22	0.25	0.75	0.01	12	20	0.3	17.3392	24.7806
23	0.25	1	0.0125	2.7	30	0.4	2.0415	6.1988
24	0.25	1.25	0.015	5	6	0.5	24.5772	27.8107
25	0.25	1.5	0.0175	7.8	10	0.1	8.6147	18.7048

Taguchi technique produces several trials depending on the control variables and their levels. Furthermore, this approach transforms the response output data to a signal-to-noise (S/N) ratio. Then, the signal represents the desired value, whereas the noise is the unwanted standard deviation from the mean value. The log transformation of mean square deviation (MSD) calculates the S/N ratio Eq. (21). MSD is considered a more effective tool for comparison than conventional measurements. It may determine S/N ratios in three ways: Larger is better, nominal is better, and smaller is better. If the output response needs to be maximized, the larger is better is used^38^. When attempting to attain current S/N ratios, the nominal is better condition is employed. The S/N ratio was estimated as an Eq. (21). In which *y*_*i*_ is the trial response obtained by the simulation and *n* is the number of replications for each trial. The S/N is computed as listed in Table 10 for only one reproduction for each trial (*n* = *1*).

21 $$S/N = - 10 \log \left[ {\mathop \sum \limits_{i = 1}^{n} \frac{1}{{y_{i}^{2} }}} \right]$$

Considering that “greater is the better” is adopted in the current study. Table 11 summarizes the response table of the S/N ratio for the different levels of each parameter. Setting the highest value of the S/N ratio and determining the optimal combination of parameters to provide the largest storage capacity will result in the optimal level for each parameter.

**Table 11:** Response for signal-to-noise ratios. (Larger is better).

Level	D/Hc	L/Hc	d/H	Density ratio	Time	Hp/Hc
1	− 15.4915	**1.1349**	0.8700	− 9.1343	**7.9756**	− 15.5055
2	− 3.4631	1.0769	1.0209	− 1.7475	3.8480	− 3.4721
3	3.5352	0.8599	0.6499	3.4220	0.5257	3.5018
4	8.3445	0.6604	1.1256	4.6245	− 2.0109	8.4919
5	**11.8676**	1.0606	**1.1263**	**7.6280**	− 5.5459	**11.7764**
Delta	27.3591	0.4745	0.4764	16.7622	13.5215	27.2819
Rank	1	6	5	3	4	2

Significant values are in bold.

Relying on the parametric analysis results of the S/N, the optimal design parameters for the gravity storage system were identified as piston diameter ratio ($0.25{ }H_{C}$), piston height ratio ($0.5{ }H_{C}$), piston density ratio (12 $\rho_{w}$), charging or discharging time (6 min), return pipe diameter ratio ($0.01{ }H_{C}$), and the return tube length ($0.5{ }H_{C}$).

**Figure 9:**
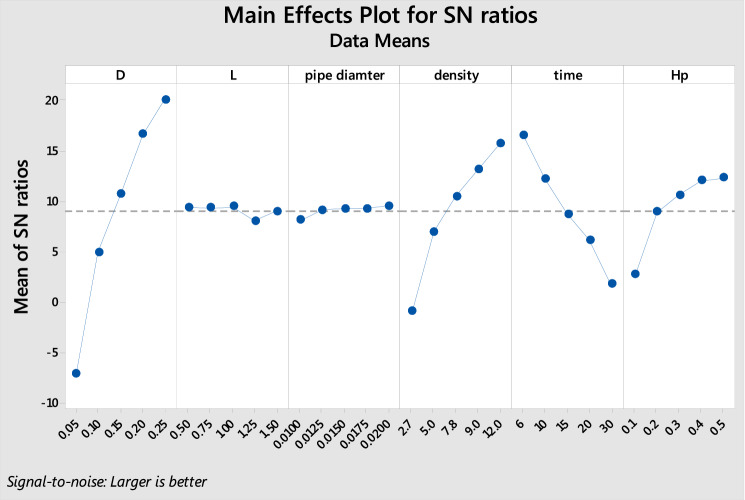
Signal-to-noise ratio on each factor.

Figure 9 also clearly shows that the piston's diameter and height significantly influence the storage capacity. In contrast, other parameters have minimal effect on power generation. Based on these findings, the piston dimension should be considered further throughout the design process. The piston density and charge/discharge time are the following influencing parameters.

The optimal combination of the parameters that is obtained from S/N ratio Graph is A5–B1–C5–D5–E1–F5 (A5 = 0.25, B1 = 0.5, C5 = 12, D5 = 6, E1 = 0.01, and F5 = 0 0.5), which requires a confirmation test.

ANOVA of the data was done using Minitab Software for hydraulic power to analyze the influence of (A) piston diameter ratio, D/H_c_, (B) Return pipe length ratio, L/H_c_, (C) Return pipe diameter ratio, d/H_c_, (D) Piston specific density, (E) Charging / discharging time, and (F) Piston Height ratio, Hp/H_c_. ANOVA allows the analysis of each control factor influence on the total variance of the results.

**Figure 10:**
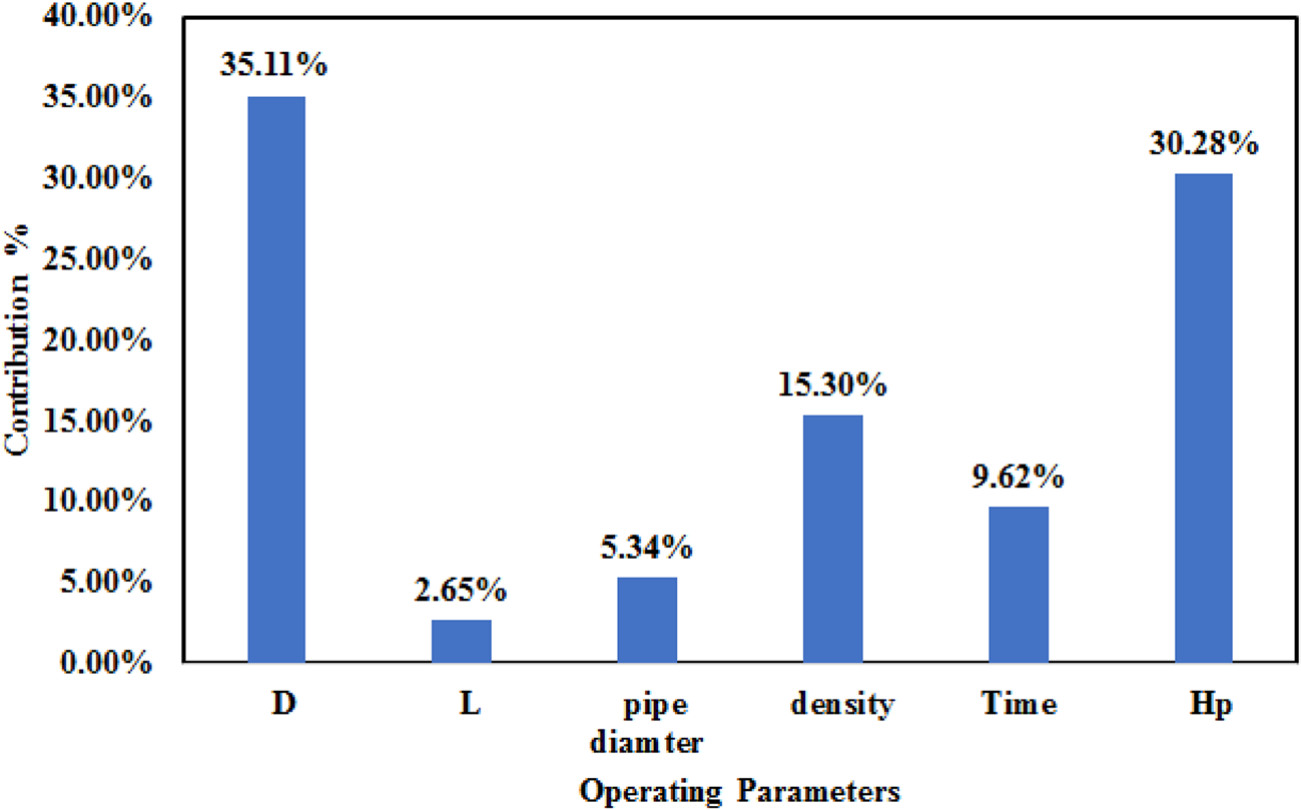
Contribution percentage of each parameter on the variation of storage capacity.

The ANOVA of the hydraulic power is shown in Table 12. According to Fig. 10, the ANOVA results showed that piston diameter, height, density ratio, and charging/discharging time have a significant impact of 35.11, 30.28, 15.30, and 9.62%, respectively. Moreover, the significant effect of these four parameters can be noticed by investigating F-value and *P* value. The *P* value is < 0.05 indicates the statistically significant impact of these parameters. The same remark relies on the F-value (select high positive value). The effects of the other two parameters on the energy storage capacity are minor and can be considered insignificant. It is clear from the table that the maximum error value is 1.7%, which confirms the validity of the results.

**Table 12:** Analysis of variance (ANOVA) of gravity energy storage.

Source	DOF	Adj SS	Adj MS	F-value	*P* value	% Contribution
Piston diameter	1	637.18	159.29	34.27	0	35.11
Return pipe length	1	48.01	12	0.91	0.353	2.65
Return pipe diameter	1	96.85	24.21	0	0.983	5.34
Density ratio	1	277.69	69.42	12.14	0.003	15.30
Charging/recharging time	1	174.66	43.66	8.5	0.009	9.62
Piston height	1	549.42	137.36	25.46	0	30.28
Error	18	30.8502	18.017	7.71168		1.70
Total	24	1814.72				100.00

## Conclusion

This study aimed to provide a parametric analysis of gravitational energy storage systems. MATLAB Simulink was used to generate the system's model then the Taguchi method was used to optimize these design parameters. The six studied parameters were the piston diameter and height, the return pipe length and diameter, the piston relative density, and the charging/discharging time. The ANOVA method showed that the piston diameter, height, density ratio, and charging/discharging time have percentage impacts of 35.11, 30.28, 15.30, and 9.62%, respectively, on the system performance. In addition, the pipe parameters (length and diameter) have a relatively minor impact on the power. The optimal combination of the investigated parameters has also been determined. The optimal threshold for each factor was identified as the Piston diameter (0.25 × H_C_), piston height (0.5 × H_C_), piston density (12 × water density), charge or discharge time (6 min), return pipe diameter (0.01 × H_C_), and returns pipe length (0.5 × H_C_), where H_C_ is the container height. The results of the current research can be utilized as design guidelines for gravity energy storage devices in future studies. From the perspective of this work, the optimal combinations of the parameters will be used to build an actual energy storage prototype.

## List of symbols

*A*: Piston area (m^2^)
*Q*: Flow rate (m^3^/s)
$Q_{A}$: Flow rate above the piston (m^3^/s)
$Q_{B}$: Flow rate below the piston (m^3^/s)
*P*: Pressure (Pa)
$P_{A}$: Pressure above the piston (N/m^2^)
$P_{B}$: Pressure below the piston (N/m^2^)
$E_{W}$: Bulk modulus of water (N/m^2^)
*x*_*p*_: Piston position (m)
$V_{A}$: Volume above the piston (m^3^)
$V_{B}$: Volume below the piston (m^3^)
*m*: Piston mass (kg)
$P_{hyd - turbine}$: Turbine hydraulic power (watt)
$S/N$: Signal-to-noise ratio
*N*: Total number of trials
*n*: Number of replications for each trial
*DOF*: Degree of freedom
$$H_{C}$$: Contained height (m)
$$H_{P}$$: Piston height (m)
$d$: Diameter of the piston (m)
$\dot{x}_{P}$: Velocity of the piston (m/s)
$\ddot{x}_{P}$, $a_{P}$: Acceleration of the piston (m/s^2^)
$${\text{Re}}$$: Reynolds number
$\mu$: Water dynamic viscosity(kg/ms)
$\rho$: Water density (kg/m3)
$v$: Velocity through the pipe (m/s)
$$d$$: Pipe diameter (m)
*f*: Pipe friction factor
$$h_{l,}$$: Head loss (m)
$$H_{T}$$: Total head (m)
*y*: The response
*M*: Average performance
*MS*: Mean squares
*SS*: Sum of squares
